# Treatment of Dairy Industry Wastewater and Crop Irrigation Water Using AgBr-Coupled Photocatalysts

**DOI:** 10.3390/nano15110848

**Published:** 2025-06-02

**Authors:** M. Hernández-Laverde, J. J. Murcia, J. A. Navío, M. C. Hidalgo, F. Puga

**Affiliations:** 1 Grupo de Catálisis, Universidad Pedagógica y Tecnológica de Colombia (UPTC), Avenida Central del Norte, Tunja 150002, Boyacá, Colombia; monica.hernandez06@uptc.edu.co; 2 Grupo de Investigación Agroalimentaria (GIA), Escuela de Ciencias Básicas Tecnología e Ingeniería, Universidad Nacional Abierta y a Distancia (UNAD), Calle 5 #1-08, Sogamoso 15221, Boyacá, Colombia; 3 Instituto de Ciencia de Materiales de Sevilla (ICMS), Centro Mixto Universidad de Sevilla-CSIC, Américo Vespucio 49, 41092 Seville, Spain; navio@us.es (J.A.N.); carmen.hidalgo@csic.es (M.C.H.); 4 Instituto de Ciencias Agro-Alimentarias, Animales y Ambientales (ICA3), Campus Colchagua, Universidad de O’Higgins, Ruta I-90 Km 3, San Fernando 3070000, Chile; felipe.puga@uoh.cl

**Keywords:** AgBr, coupled photocatalysts, polluted river, dairy wastewater

## Abstract

This work describes the application of three different AgBr heterojunctions with TiO_2_, SnO_2_ and WO_3_ in the treatment of two water sources: wastewater from a dairy industry facility (WDI) and water from a polluted river (WPR). All heterojunctions were widely characterised, and it was observed that the physicochemical properties of all the coupled materials were similar; however, the highest elimination of Enterobacteriaceae (>90%) was obtained with the AgBr/WO_3_(20%) photocatalyst in WDI. Under the same conditions, with this photocatalyst, the complete removal of bacteria (i.e., *E. coli*, total coliforms and other Enterobacteriaceae) was achieved in WPR. The chlorides, hardness and colour in the two water samples decreased after photocatalytic treatment with all the coupled materials. However, nitrate levels and chemical oxygen demand increased due to the possible formation of intermediary species from the photodegradation of organic pollutants and the release of metabolic intermediates from bacterial degradation during the photocatalytic process. Overall, heterogeneous photocatalysis based on AgBr-coupled materials shows potential as a tertiary treatment for WDI and for the purification of vegetable irrigation water. However, it is still important to consider the need to optimise the integrity of photocatalytic materials in order to maintain their bactericidal effectiveness through continuous reuse.

## 1. Introduction

The application of advanced oxidation technologies (AOTs) for environmental remediation has been extensively studied in recent years, and heterogeneous photocatalysis has emerged as one of the most promising AOTs for the degradation of organic pollutants, disinfection and the removal of toxic metals from aqueous media [1]. In brief, the photocatalytic process occurs through the generation of reactive oxygen species (ROS) induced by UV–Vis irradiation in a semiconductor, which participate in redox reactions with the organic compounds present in the reaction medium [2,3]; these oxidant species are responsible for the degradation of pollutants and the removal of bacteria [4,5].

This reaction mechanism based on ROS has led to titanium dioxide (TiO_2_) being the most studied photocatalyst, mainly for water and air treatment and disinfection [6,7,8]; this material has well-known properties suitable for photocatalytic processes, such as availability, low toxicity and a low cost [2,9]. However, TiO_2_ presents some disadvantages, such as its absorption being limited to UV light and the fast recombination of photogenerated charges [10,11,12,13]. In order to overcome these disadvantages, other semiconductors have been studied as photocatalysts [11,13]. Thus, for example, ZnO [14], SnO_2_ [15,16], WO_3_ [17,18] and AgBr [19,20,21] have been reported to be efficient photocatalytic materials for the degradation of some organic pollutants.

In this context, SnO_2_ presents interesting electrical, optical and optoelectronic properties, as well as high stability for photocatalytic applications. Nevertheless, its high band-gap value (3.6 eV) limits its use in solar applications [22]. On the other hand, tungsten trioxide is an n-type semiconductor with some advantages, such as its non-toxic nature, moderate cost and low band gap, ranging from 2.4 to 2.8 eV, favouring light absorption in the visible region [23]. Silver bromide is also a well-known n-type photosensitive semiconductor, which is characterised by its narrow band gap (2.5–2.6 eV), its high photocatalytic activity in the visible region and its bactericidal effect. Furthermore, due to the suitable position of its conduction and valence bands, AgBr has been widely studied for the development of coupled materials with type II heterojunctions or Z-scheme systems [24].

In order to evaluate all these materials as individual or coupled photocatalysts, many researchers worldwide have studied their effectiveness in the degradation of different organic pollutants [25,26,27,28] and in bacteria removal [29]. Most of these investigations have used laboratory-prepared model solutions of different pollutants and isolated bacterial strains [30,31,32]. These studies represent an interesting starting point to understand the behaviour of photocatalytic materials under ideal laboratory conditions. However, these circumstances are very different from the real configurations present in industrial effluents or in real sources of contaminated water, where different pollutants and bacteria are usually present concurrently. Therefore, the behaviour of a photocatalytic material can be radically different from that observed in the degradation of individual pollutants. For this reason, it is very relevant to test photocatalysts by using real effluents as substrates once their efficiency has been tested on a laboratory scale, as has been reported in different works [33,34,35].

Taking into account this overview, in our research group, some materials based on AgBr bare and coupled with TiO_2_, WO_3_ and SnO_2_ have been developed and investigated firstly as photocatalysts for lab-prepared solutions of model molecules of different pollutants; from these studies, it was possible to obtain high efficiency in the degradation of rhodamine B, caffeine and caffeic acid as model pollutants [36,37,38]. Taking these results into account, the next experimental step and the main objective of the present work were focused on the evaluation of these AgBr composite photocatalysts in the treatment of two real water sources, which contain many different pollutants: (i) wastewater samples from an industrial dairy production facility; (ii) a Colombian river highly polluted with industrial and domestic wastewater.

The first water source selected for this study was mainly chosen because dairy production is a relevant industry in different Latin American countries [39]. The discharge of dairy industry effluents directly into drains, rivers or nearby soils without prior treatment negatively impacts the environment [40]; due to their high concentrations of organic matter, the discharge of these waters into aquatic systems can reduce dissolved oxygen, harming the survival of different species in these ecosystems [41]. Although the organic pollutants present in these effluents can be biodegradable, this process is not significant because it is extremely slow [42]. In addition, the second water source evaluated in the present work represents rivers contaminated with wastewater, which are generally used to irrigate crops, allowing certain microorganisms, such as enteric bacteria, to remain in vegetables and other crops for human or animal consumption [43,44]. These bacteria are responsible for a variety of diseases, especially in children in developing countries [44,45,46,47].

In order to evaluate the effectiveness of the AgBr heterojunctions with TiO_2_, SnO_2_ and WO_3_ in the treatment of different pollutants present concurrently in the water source, in this work, we analysed different water quality control parameters, such as nitrates, chlorides, total hardness and bacterial concentration, and the results obtained are described in detail in this manuscript.

A bibliometric analysis on the synthesis and evaluation of coupled/heterojunction photocatalytic materials gives us information on different systems, such as Ag/AgBr/TiO_2_ [48,49], AgBr/TiO_2_ [38,50], TiO_2_/SnO_2_ [51], AgBr/WO_3_ [36,52], Ag−AgCl/WO_3_ [53], Ag/AgCl [54] and AgBr/SnO_2_ [37]. Different authors have reported interesting and valuable results on these materials, which highlighted their effectiveness in the degradation of different organic compounds and in the elimination of *E. coli*. From these reports we can indicate that the novelty of combining AgBr with TiO_2_, SnO_2_ and WO_3_ in our work lays in the fact that to date, and to our knowledge, no reports about industrial wastewater treatment and bacteria elimination using these coupled materials are found in the scientific literature. Therefore, the results described in this study represent an interesting starting point for further applications of AgBr-based photocatalysts as an alternative for water and wastewater treatment.

## 2. Materials and Methods

### 2.1. Photocatalytic Material Preparation

All samples, pristine and heterojunctions, were prepared according to the following synthesis procedure [36,37,38], developed by our research group. 

TiO_2_: Faceted TiO_2_-{001} was obtained by mixing Ti (IV) isopropoxide with a commercial aqueous HF solution (40% *v*/*v*), using a volume ratio of Ti precursor/HF of 25/4 *v*/*v*; then, the resulting solution was hydrothermally treated at 200 °C for 24 h. Subsequently, the solid was filtered, washed with water and then dried at 100 °C for 24 h.

WO_3_: An aqueous solution of HCl at 8 M and Na_2_WO_4_. 2H_2_O was prepared and hydrothermally treated at 100 °C for 24 h. The solid obtained was filtered, washed and dried, and then calcined at 400 °C for 4 h.

SnO_2_: SnCl_4_.5H_2_O was dissolved in 40 mL of water, stirred for 30 min and then hydrothermally treated at 190 °C for 12 h; then, the powder was filtered, washed with water and dried at 100 °C for 24 h.

AgBr: An aqueous solution of 50 mL with a certain amount of KBr was prepared and added dropwise to another aqueous solution of 50 mL of AgNO_3_ (molar ratio of Ag:Br = 1:1); the resulting mixture was stirred for 2 h in the dark. Subsequently, the solid was filtered, washed with water and then dried at 100 °C for 24 h.

AgBr-coupled photocatalysts: The composite materials were obtained by the precipitation–deposition of AgBr over the TiO_2_, WO_3_ or SnO_2_ already synthesised. For this purpose, the same procedure followed for AgBr synthesis was used, but in this case, a certain amount of the TiO_2_, WO_3_ or SnO_2_ synthesised was added to the AgNO_3_ solution, before mixing it with the KBr solution. The amount of metal oxide (TiO_2_, WO_3_ or SnO_2_) in the AgNO_3_ solution was added according to different values of the AgBr/metal oxide ratio (100 mol AgBr/X mol metal oxide or alternatively X* mol AgBr/100 mol metal oxide). Thus, the prepared hybrid samples will be hereafter referred to as AgBr/metal oxide(X%) or AgBr(X*%)/metal oxide. 

In a previous work [38], we studied the coupling of AgBr(X%)/TiO_2_-faced in three different AgBr molar ratios (X = 10, 20 and 50). In that work, on the basis of a comprehensive study, it was concluded that the AgBr(50%)/TiO_2_ sample presented better results in terms of successive reuse cycles and the mineralisation of RhB dye. Similarly, in two other works, the coupled materials AgBr/WO_3_(20%) [36] and AgBr/SnO_2_(20%) [37] allowed us to achieve the highest degradation and mineralisation of RhB. Following the results reported in the mentioned references, we prepared the AgBr-coupled photocatalysts by combining the materials which present the best performance in the degradation of different organic pollutants. Thus, we decided to synthesise AgBr(50%)/TiO_2_, AgBr/WO_3_(20%) and AgBr/SnO_2_(20%).

### 2.2. Physicochemical Analysis of AgBr-Coupled Materials

An X’Pert Pro PANalytical X-ray diffractometer equipped with Cu X-ray source radiation Kα (40 mA, 45 kV) was employed for XRD analysis, and the obtained patterns were fitted by using a Voigt function.

A Micromeritics ASAP 2010 instrument was used for S_BET_ measurements; these analyses were carried out at low temperature (77 K) with N_2_ adsorption, and before the measurements, the samples were degasified at 150 °C for 30 min under helium flow.

The UV–Vis DRS spectra were collected with a Varian model Cary 100 spectrophotometer equipped with an integrating sphere. The indirect optical band-gap value (Eg) of the synthesised materials was determined by Tauc plots [55]. The valence band (VB) and conduction band (CB) positions of AgBr, TiO_2_, WO_3_ and SnO_2_ were estimated by using the following empirical equations [56,57]:

$$E_{\mathrm{VB}}=X\;-{\;E}^{e}+0.5E_{g}$$

$$E_{\mathrm{CB}}=E_{\mathrm{VB}}\;-\;E_{g}$$

where the following apply:

$E_{\mathrm{VB}}$ and $E_{\mathrm{CB}}$ are the valence and conduction band potentials.

$E^{e}$ is the energy of free electrons on the hydrogen scale (i.e., 4.5 eV).

X is the electronegativity of each semiconductor (i.e., AgBr = 4.42 eV [58]; TiO_2_ = 5.81 eV [59]; WO_3_ = 6.33 eV [60]; SnO_2_ = 6.25 eV [61]).

TEM images were obtained with an F200S FEI microscope Talos™ (Thermo Fisher Scientific—Materials Science, Hillsboro, OR, USA).

The XPS analyses were carried out on a Photoelectron Spectroscopy System, “SPECS” PHOIBOS MOD: 150 MCD (SPECS Group, SPECS Surface Nano Analysis GmbH, Berlin, Germany), at 50 eV path energy and at a pressure below 2 × 10^−9^ Torr.

### 2.3. Water Sampling and Analysis

Two water sources were analysed. The first source was the wastewater from the outflow of a dairy industry located at the following geographic coordinates: 5.67260, −72.99210. In order to adhere to industrial regulations, the name of this local industry is not indicated in this manuscript, but its main activity is related to the production of cow milk derivatives. The second sample was taken from a Colombian river called “Jordán”, which is highly polluted by industrial and domestic wastes; this water source is commonly used for vegetable irrigation in fields bordering the river (geographic coordinates: 5.689573, −72.980619).

Water samples were collected following the protocols described in the Standard Methods for the Examination of Water and Wastewater [62]. As it was previously indicated, in this work, real water samples were evaluated under the realistic conditions found during the sampling process. These samples can present different composition depending on environmental factors such as pluviosity; therefore, in order to obtain representative results about the composition of the samples, the river water was sampled at two different times of the year.

After sampling, the water was kept under cool conditions during transport and storage before analysis. Then, each water source was physicochemically analysed for chemical oxygen demand (COD), chlorides, nitrates, pH, total hardness, calcium hardness and real colour. These analyses were performed with a Spectroquant^®^ Move 100 instrument (Merck KGaA, Darmstadt, Germany), and to ensure the reproducibility of results, each assay was performed twice.

Microbiological analysis was carried out by the membrane filtration method (Merck SM 9222B [62]), where Chromocult^®^ agar (Merck KGaA, Darmstadt, Germany) was employed as the culture medium for coliforms species, and the bacterial concentration in the samples was reported as Colony-Forming Units (CFU)/100 mL.

Table 1 summarises the analyses performed in this work and the maximum permissible limits on selected parameters reported in the Colombian regulations for (i) water discharges in the dairy production industry (Resolution 631/2015 [63]) and (ii) crop irrigation water (Resolution 1207/2014 [64]). According to Colombian regulations, for some of these parameters, no maximum limit is established, and only their analysis should be reported (“Analysis”), while other analyses are not required (“NR”).

### 2.4. Photocatalytic Treatment of Water Sources

The photocatalytic performance of the AgBr-coupled photocatalysts in the treatment of the two water sources was evaluated with a discontinuous Pyrex batch reactor, illuminated by an Osram Ultra-Vitalux lamp (300 W; Osram, Munich, Germany) with a sun-like radiation spectrum and a main line in the UVA range at 365 nm. The intensity of the UVA light was fixed at 120 W/m^2^. A total of 250 mg of photocatalyst was added to 250 mL of wastewater inside the reactor (1 g/L dosage). After 5 h of treatment under continuous stirring, illumination and 0.84 L/h of air bubbling (as the oxygen source, which was employed as the oxidant agent), the tested photocatalysts were recovered by filtration, and the treated water was analysed by the physicochemical and microbiological methods previously described in Section 2.3.

All photocatalytic tests (standard deviation of 0.05) and the water quality control parameter analysis were carried out twice, and the values reported in this manuscript are those estimated as the arithmetic average.

It is important to say that in order to achieve the adsorption–desorption equilibrium between wastewater and the photocatalyst, each photocatalytic test started with stirring in the dark for 20 min, and after this time, the lamp was switched on.

## 3. Results and Discussion

### 3.1. Physicochemical Properties of AgBr-Coupled Materials

**Figure 1:**
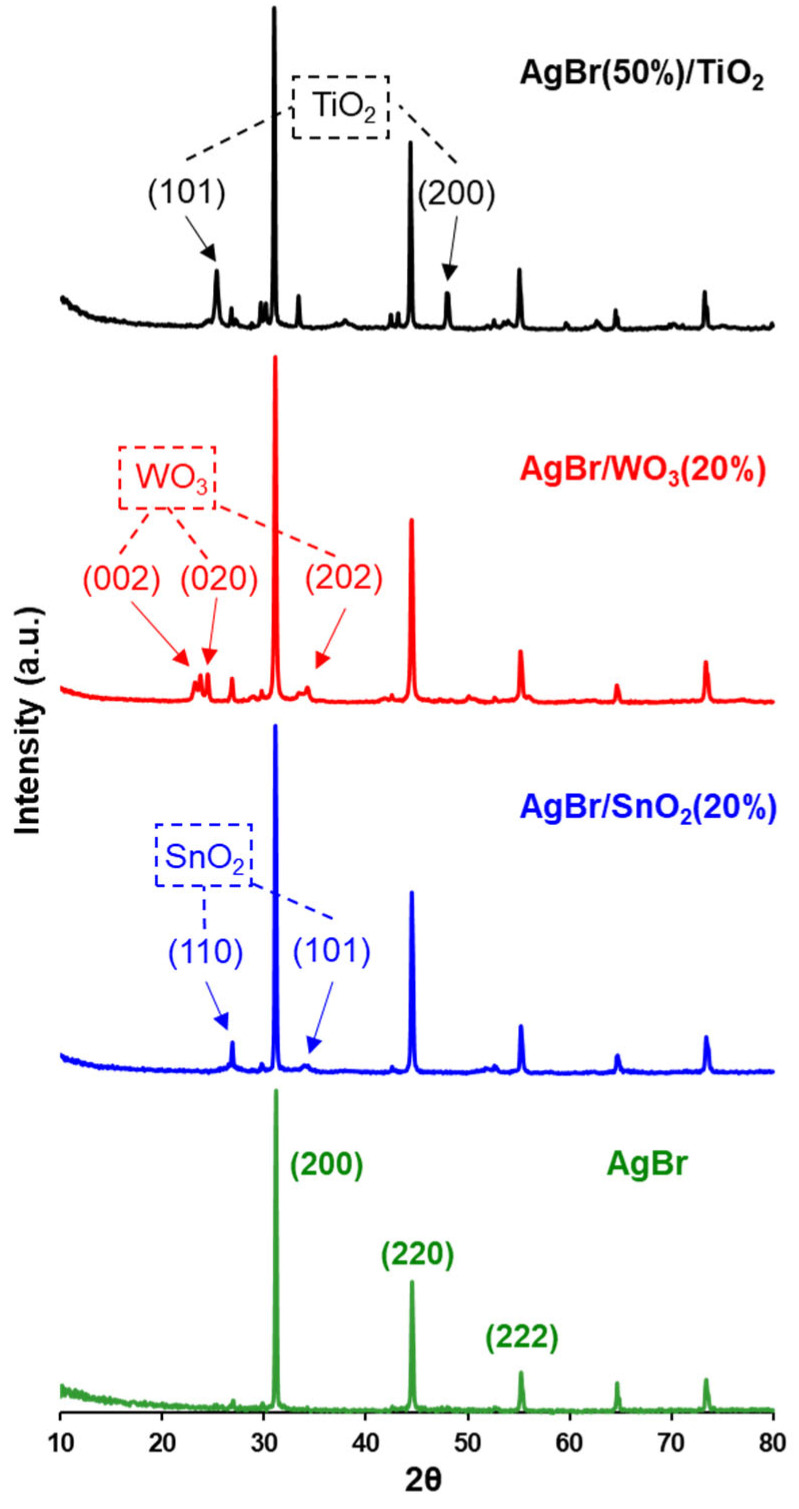
X-ray diffraction patterns of AgBr and coupled photocatalysts. The Miller indexes (hkl) indicated in each diffractogram correspond to the main patterns of AgBr, TiO_2_, SnO_2_ and WO_3_.

The XRD patterns of the synthesised photocatalytic materials are plotted in Figure 1. In this figure, the characteristic peaks corresponding to the cubic structure of AgBr can be seen (JCPDS No. 00-001-0950), located at the 2Ɵ values of 31.2°, 44.9° and 55.1° and associated with the (200), (220) and (222) crystalline planes, respectively.

For the AgBr(50%)/TiO_2_ photocatalyst, the characteristic peaks of TiO_2_ anatase (JCPDS No. 00-021-1272) are shown in Figure 1; these signals are located at the 2Ɵ degrees of 24.8° and 48.1°, which correspond to the (101) and (200) planes. As expected, in this material, the peaks corresponding to AgBr present the lowest intensity of the series of materials analysed; this is due to the lower AgBr molar ratio used in the synthesis of this material.

For the AgBr/WO_3_(20%) photocatalyst, all diffraction peaks corresponding to the monoclinic crystalline phase of WO_3_ (JCPDS No. 00-043-1035) are evidenced in Figure 1. Finally, in the same figure, for the AgBr/SnO_2_(20%) material, the peaks of tetragonal SnO_2_ (JCPDS No. 00-021-1250) are visualised, attributed to the (110) and (101) crystalline planes and located at the 2Ɵ values of 26.8° and 34.2°, respectively.

The average crystallite size of AgBr in each photocatalyst was also determined by XRD data by using the Scherrer equation, and the results obtained are reported in Table 2. As can be seen, in this table, the AgBr crystallite size (175.5 nm) is larger when it is coupled with titania (242.4 nm) and decreases when coupled with SnO_2_ (149.9 nm) or WO_3_ (107.1 nm). These changes observed in the AgBr crystallite size could be due to the deformations produced in each coupling, which are related to contact stresses and stacking failures, which can relatively modify the shape and position of the XRD patterns [65].

**Figure 2:**
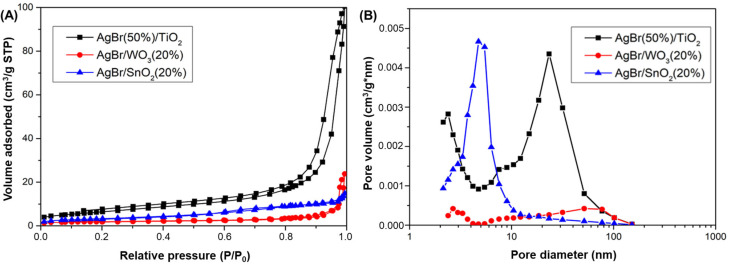
N_2_ adsorption–desorption isotherms (**A**) and pore size distribution (**B**) of AgBr-coupled photocatalysts.

The BET specific surface areas of all samples are compiled in Table 2. These results indicate that all composite materials have a lower surface area than the pristine metal oxides, which indicate that the coupling with AgBr leads to a decrease in the surface area. The N_2_ adsorption–desorption isotherms of the coupled systems are provided in Figure 2A. As can be seen, the composites exhibit a type IV isotherm according to the IUPAC classification, with a H_3_ hysteresis loop (observed in the P/P_0_ range of 0.80–0.95), with the knee at P/P_o_ equal to 0.2, thus indicating that these samples have a mesoporous texture with slit-like pores [66]. In this case, the initial monolayer–multilayer adsorption on the mesopore walls, which takes the same path as the corresponding part of a type II isotherm, is followed by pore condensation [67].

On the other hand, the Barrett–Joyner–Halenda (BJH) model was also used to evaluate the pore size distribution of the composite photocatalysts, and the results are plotted in Figure 2B. As it can be observed, the coupled materials have a bimodal pore diameter distribution, where the highest pore concentrations in AgBr/SnO_2_(20%) and AgBr(50%)/TiO_2_ are found as 2–8 and 10–50 nm, respectively, while for AgBr/WO_3_(20%), its pore volume is much smaller than for the other two AgBr-based photocatalysts.

**Figure 3:**
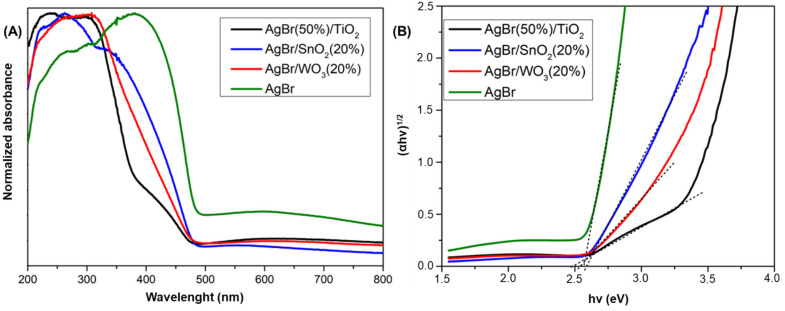
UV–Vis diffuse reflectance spectra (**A**) and Tauc plots (**B**) for AgBr-coupled materials. Dotted lines indicate intersection points between axes, where band gaps were calculated.

Figure 3 shows the absorption spectrum and the Tauc plots for pristine and coupled AgBr materials. For AgBr it is possible to identify in this figure its characteristic absorption band between 270 nm and 500 nm. After coupling, the absorption range decreases significantly, resulting in a shift in the spectrum to the left. Moreover, there is more than one band gap for each sample. The overall absorption trend in the visible region decreases as follows: AgBr > AgBr/SnO_2_(20%) > AgBr/WO_3_(20%) > AgBr(50%)/TiO_2_. The Eg of the coupled materials (pseudo band-gap values; Table 2) are very similar and closer to the pristine AgBr, with no significant differences.

**Figure 4:**
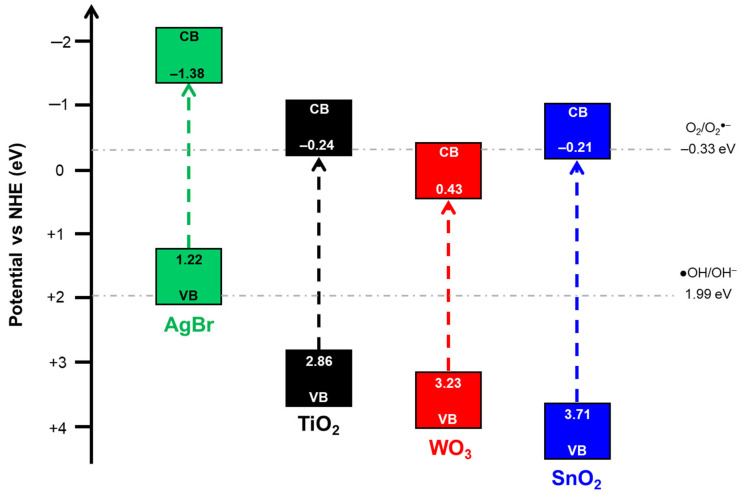
Band structures of AgBr, TiO_2_, WO_3_ and SnO_2_.

As indicated in the experimental section, the band-gap values were used to determine the VB and CB edges of the pristine photocatalysts (Table 3), and the results are presented in Figure 4. According to the edges of conduction and valence bands, only photoexcited electrons in the CB of AgBr can reduce O_2_ to form O_2_^●−^ radicals (−0.33 eV) [68]. On the other hand, the VB of the metal oxides are more positive than the standard ^●^OH/OH^−^ potential (1.99 eV) [68], thus indicating that the OH groups adsorbed on the surface of these photocatalysts can be oxidised to form ^●^OH. Nevertheless, the coupling of AgBr with any of the three photocatalysts could lead to the formation of a type II heterojunction according to the band positions. Therefore, in these coupled systems, the photogenerated electrons in the CB of AgBr could migrate towards the CB of TiO_2_, SnO_2_ or WO_3_. Similarly, the photogenerated holes in the VB of TiO_2_, SnO_2_ (only under UV light) or WO_3_ could migrate towards the VB of AgBr. Thus, by separating the charge carriers, the recombination would be delayed in both semiconductors, increasing the photocatalytic activity.

**Figure 5:**
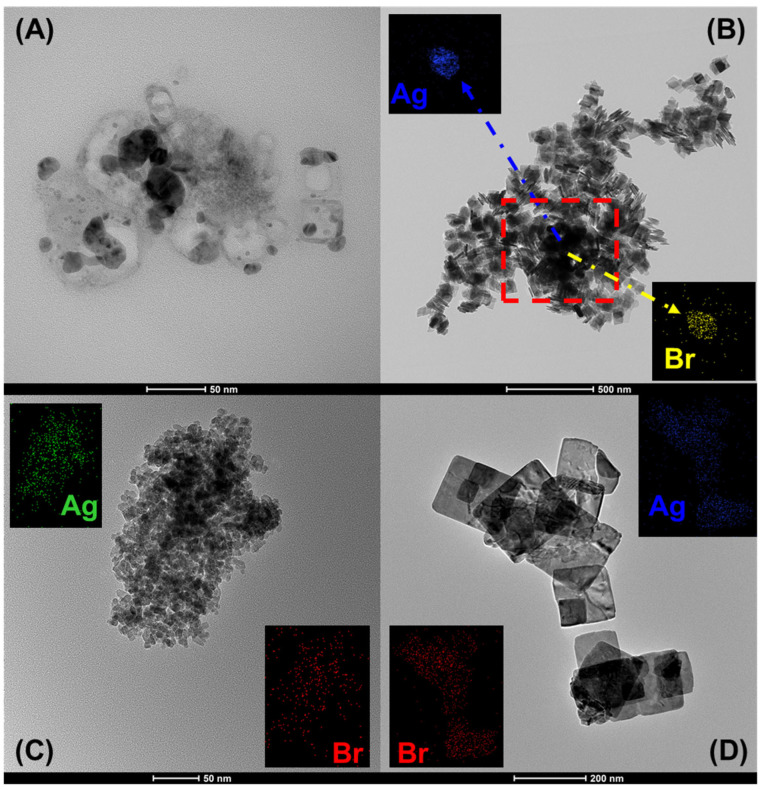
Selected TEM images of analysed photocatalysts. (**A**) AgBr, (**B**) AgBr(50%)/TiO_2_, (**C**) AgBr/SnO_2_(20%) and (**D**) AgBr/WO_3_(20%).

Figure 5 shows selected TEM images of pristine AgBr and the coupled samples. The obtention of TEM images of AgBr was very difficult, as its particles can be broken by the high-energy electron beam used in this analysis [69]. For this reason, the TEM images of each coupled material include the elemental mapping of Ag and Br to identify the AgBr particles in the hybrid materials.

For AgBr (Figure 5A), ellipsoidal and spherical particles are observed, with sizes between 5 and 30 nm. For AgBr(50%)/TiO_2_ (Figure 5B), TiO_2_ platelets with a size of 100 nm are observed frontally or laterally together with spherical or elliptical AgBr particles of different sizes. In the case of AgBr/SnO_2_(20%) (Figure 5C), the magnification of this image reveals numerous highly agglomerated nanorods. A detailed examination of these images, using the magnification of the micrographs, revealed two particle sizes: larger particles corresponding to AgBr and smaller particles corresponding to SnO_2_.

On the other hand, for the AgBr/WO_3_(20%) sample (Figure 5D), it is possible to identify WO_3_ as square particles with sizes ranging between 50 and 200 nm, as well as AgBr particles with a round ellipsoidal shape with sizes between 5 nm and 25 nm. The average size of AgBr in the coupled materials was estimated by “close inspection” by using the magnification of micrographs and the reference size of micrographs. The elemental mapping images inserted in Figure 5 confirm the presence of AgBr particles together with the corresponding metal oxides in each composite material.

**Figure 6:**
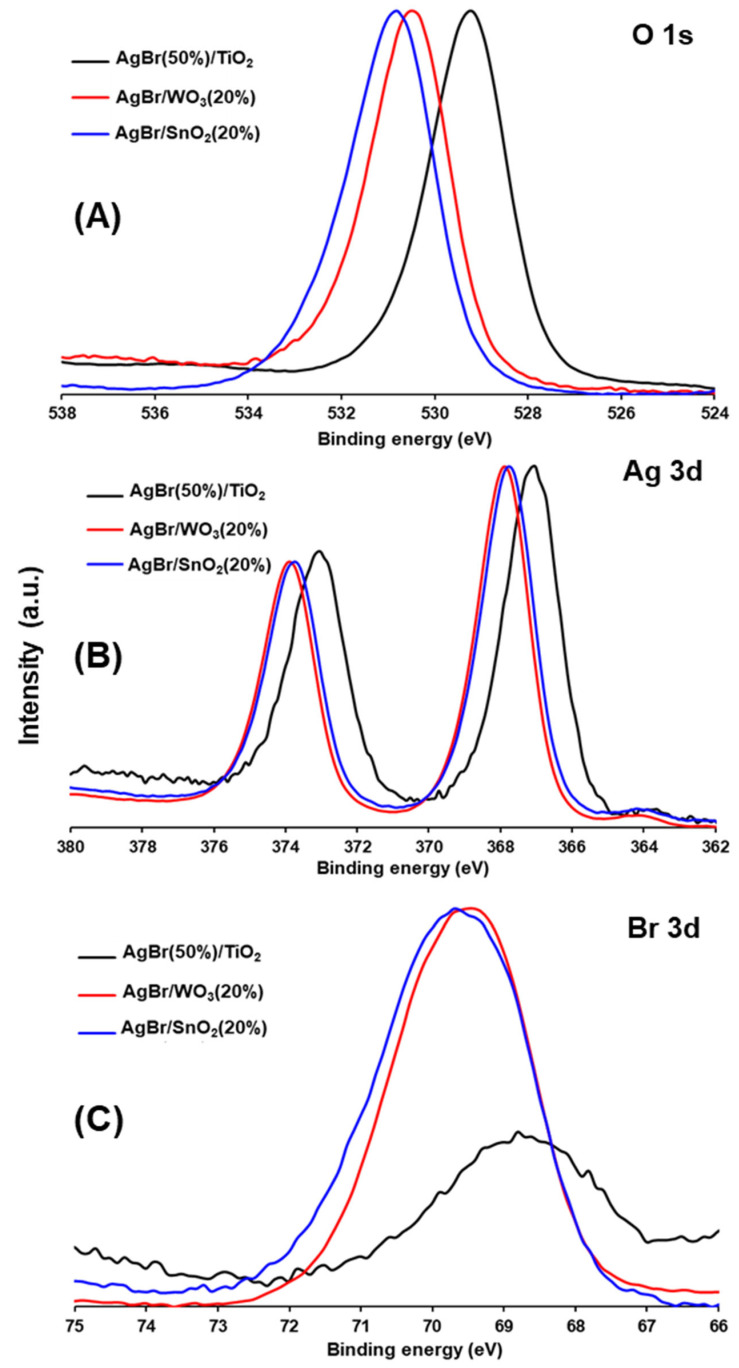
High-resolution XPS spectra of AgBr-coupled photocatalysts. (**A**) O 1s region, (**B**) Ag 3d region and (**C**) Br 3d region.

The surface elemental composition of the coupled materials was studied by XPS, and selected spectral regions (O 1s, Ag 3d and Br 3d) are presented in Figure 6.

As observed in Figure 6, the main peaks in the O 1s region of all samples (Figure 6A) evidenced some differences. For AgBr(50%)/TiO_2_, the main peak is located at 529.2 ± 0.1 eV, while for AgBr/SnO_2_(20%) and AgBr/WO_3_(20%), the peaks are at 530.8 ± 0.1 eV and 530.5 ± 0.1 eV, respectively. In every case, the O 1s signal corresponds to two components, ascribed to reticular oxygen and surface OH^−^ groups.

The Ag 3d region spectra (Figure 6B) show two well-defined peaks in all samples, corresponding to Ag 3d_5/2_ and Ag 3d_3/2_ of Ag^+^ in AgBr [70,71]. In this region, a shift to higher binding energies is observed for the AgBr/SnO_2_(20%) and AgBr/WO_3_(20%) powders, compared with the TiO_2_ coupled material.

In the case of the Br 3d region (Figure 6C), the AgBr/SnO_2_(20%) and AgBr/WO_3_(20%) samples presented similar main peaks located at 69.7 ± 0.1 eV and 69.4 ± 0.1 eV, respectively, while the AgBr(50%)/TiO_2_ sample shows a main peak located at lower binding energy (i.e., 68.8 ± 0.1 eV).

The shift to lower binding energies observed in the O 1s, Ag 3d and Br 3d regions for the AgBr(50%)/TiO_2_ material, compared with the other two composites, can be associated with the AgBr attachment to the TiO_2_ crystal surface, forming a p-n heterojunction, where the electrons could be transferred from TiO_2_ to AgBr [57].

### 3.2. Photocatalytic Treatment of Wastewater by AgBr-Coupled Photocatalysts

#### 3.2.1. Dairy Industry Effluent

Table 4 presents different parameters analysed for the dairy industry wastewater. According to Colombian regulations (Resolution 631/2015, Table 1), the industrial effluent exceeds the limit values for COD and chlorides and presents high concentrations of total hardness and total coliforms.

After photocatalytic treatment (including the photolysis-blank test) under UV–Vis illumination with the AgBr-based photocatalysts, several of these parameters significantly reduced their concentration, with a few exceptions. There were no significant changes in pH value (i.e., ±0.01), which remained practically the same before and after treatment.

**Figure 7:**
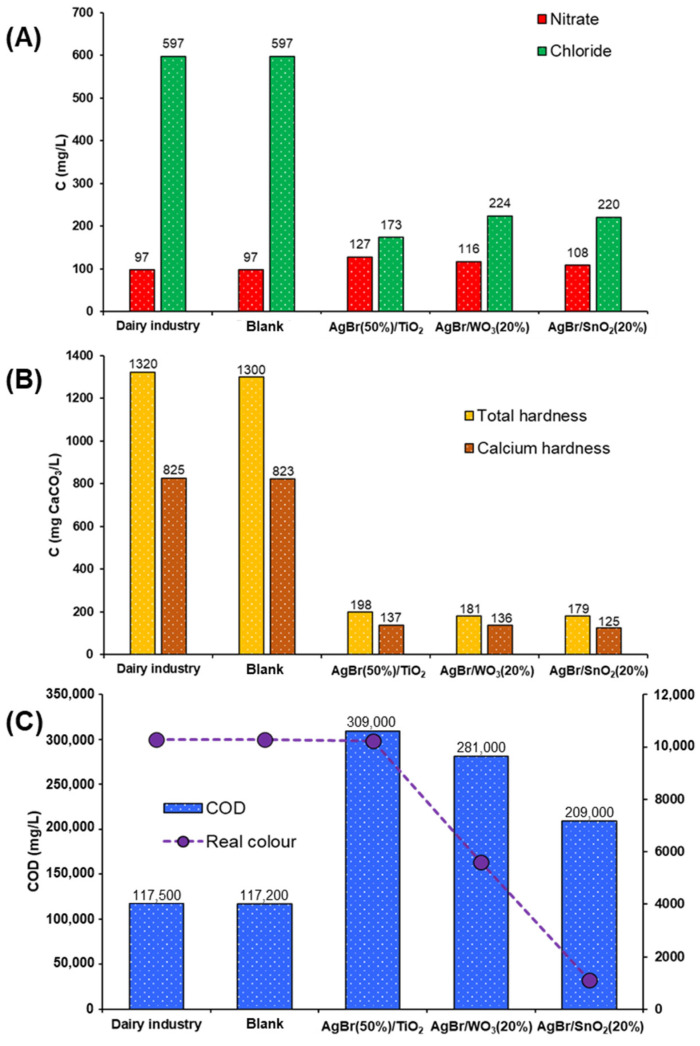
Control parameters of dairy industry wastewater before and after photocatalytic treatment. (**A**) Nitrate and chloride. (**B**) Total hardness and calcium hardness. (**C**) COD and real colour (absorbance measured at wavelength of 436 nm).

The nitrate concentration increased slightly after photocatalytic treatment (Figure 7A). This may be due to the oxidation of the nitrogen present in the pollutants of the starting water sample, together with the nitrogen compounds of the bacteria membrane after cell breakage by photocatalytic oxidation [72]. Based on these results and in order to comply with the regulations, an additional treatment for nitrate removal should be applied.

In agricultural irrigation, high chloride concentrations can significantly affect soil quality. Consequently, it is necessary to control the concentration of chlorides present in the treated water to be used for crop irrigation. Photolysis (blank test) did not affect the chloride content, while the photocatalytic process decreased considerably its concentration (Figure 7A). The adsorption of chloride ions on the surface of the photocatalysts could be the reason for this reduction. Chloride ions can also undergo a series of reactions that affect the photocatalysis attributed to hydroxyl radicals [73,74], which act as their inhibitors; it has been also observed that the decrease in O_2_^•−^ radical concentration becomes drastically large in the presence of SCN^−^ and I^−^ ions, while these decreases are small for Br^−^ and especially for Cl^−^ ions [75]. It has also been reported that hydroxyl groups and chloride ions form inner-sphere complexes with titanium ions on a TiO_2_ surface (together with water molecules), thus photogenerating ClO^−^ and H_2_O_2_, which further react to form oxygen in the singlet state with high yield [76,77].

The presence of high levels of calcium in water creates what is known as hard water, so its presence and quantity must also be analysed in water after treatment. Therefore, these parameters were also considered in our study. Similar to chlorides, total and calcium hardness concentrations also decreased after the photocatalytic process (Figure 7B). Again, the adsorption of calcium and magnesium ions on the surface of the coupled materials should be the reason of the abatement of hardness.

In order to study the adsorption of chlorides, calcium and magnesium on the coupled materials, two additional experiments using the AgBr/WO_3_(20%) photocatalyst were performed. Thus, to a commercial solution with 167.2 mg/L chloride and for a 2% *v*/*v* cow milk solution with 27.5 mg/L total hardness, 1 g/L AgBr/WO_3_(20%) was added, stirring both suspensions in the dark for 4 h. Finally, concentrations of 147.5 mg/L (commercial solution) and 17.8 mg/L (2% *v*/*v* solution) of chloride and total hardness, respectively, were obtained. These results confirm that the coupled materials present adsorption capacity for chloride, calcium and magnesium ions on their surface.

Dairy industry wastewater is a very complex sample, which may include different organic compounds such as lactose, proteins, amino acids, fat, salts, disinfectants, and alkaline and acid products; all these compounds are susceptible to be photocatalytically degraded. The COD test measures the amount of these compounds susceptible to be oxidised, whether of organic or inorganic origin. In this work, the COD concentration decreased slightly after photolysis (Figure 7C). Nevertheless, this parameter increased significantly after the photocatalytic treatment with the coupled materials, especially with the AgBr(50%)/TiO_2_ photocatalyst. Similar results were previously reported by Murcia et al. [29].

The increase in COD may be related to the damage of the bacterial cell membrane after photocatalytic treatment, which leads to the rupture and subsequent release of metabolic intermediates in the reaction medium. The photooxidation of different organic compounds present in the wastewater samples and the yielded byproducts can also contribute to the increase in the COD value [78]. Lei et al. [79], observed, by AFM, that as an effect of photocatalytic process, *E. coli* cells were broken; then, the intracellular substances seemed to be dissolved, which gives some qualitative evidence about the release of some bacterial intracellular compounds inside the reactor. The photocatalytic process led to serious damage to lipid and proteins in the bacterial cell [80], leading to bacterial inactivation; the released compounds from this process could increase the COD and, at the same time, the competition among organic matter, inactive cells, viable cells and organic pollutants present in the sample for ●OH oxidising radicals. These hypotheses are interesting starting points for further research using advanced analytical methods such as LC-MS which may include the determination of the intermediates formed during the photocatalytic treatment of dairy industry effluents, and further treatment is recommended to improve COD reduction [81,82].

In order to analyse in depth the behaviour of COD during the photocatalytic reaction, the monitoring of this parameter at different reaction times was carried out by using the AgBr/WO_3_(20%) photocatalyst. From these assays, it was observed that the COD concentration firstly increased to 306,650 mg/L after 2.5 h of photocatalytic treatment and then decreased to 281,000 mg/L at the end of the process (5 h). This indicates that COD reaches a maximum value during photocatalytic treatment; then, its concentration starts to decrease. Therefore, the photocatalytic treatment of this wastewater sample generates new species that cause an increase in COD; then, these compounds are mineralised, leading to a decrease in COD concentration.

Contrary to the COD behaviour, the real colour decreased with the coupled material treatment, but only with two composites, AgBr/WO_3_(20%) and AgBr/SnO_2_(20%), with the latter being the most effective (Figure 7C).

#### 3.2.2. Jordán River

**Figure 8:**
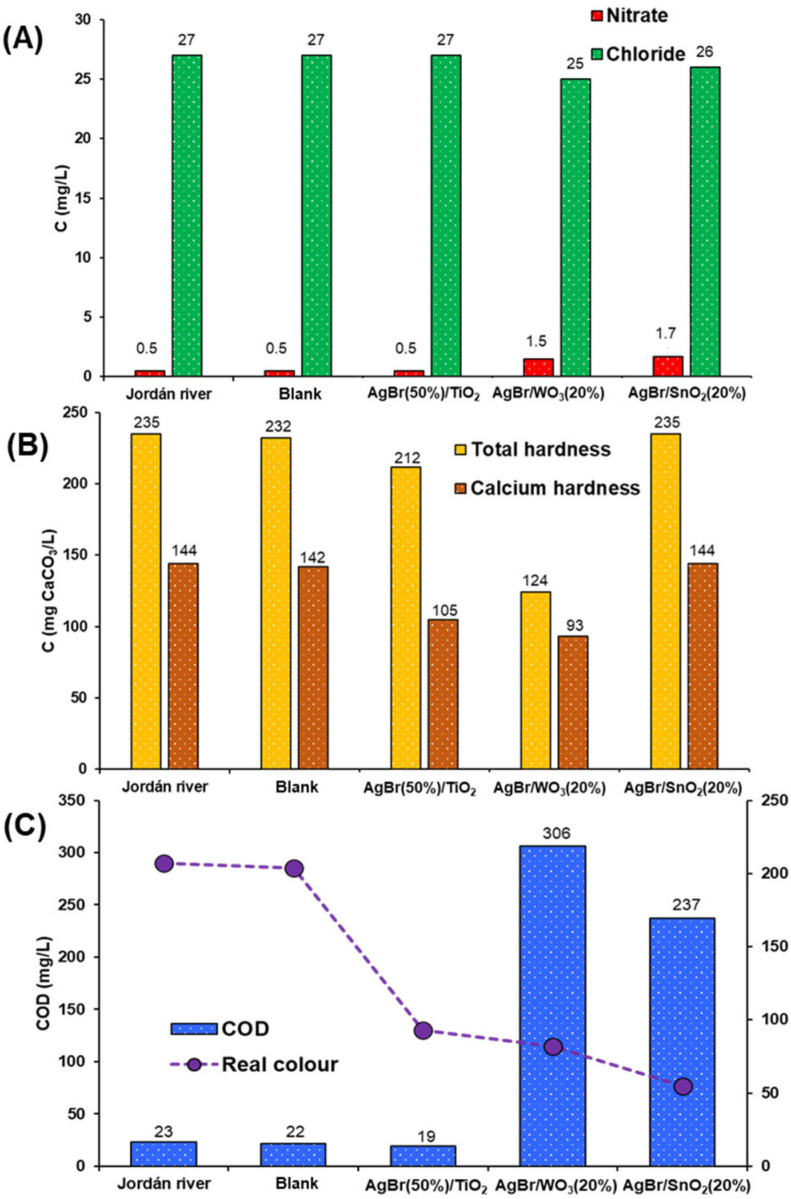
Control parameters of Jordán river sample before and after photocatalytic treatment. (**A**) Nitrate and chloride. (**B**) Total hardness and calcium hardness. (**C**) COD and real colour (absorbance measured at wavelength of 436 nm).

Table 4 summarises the control parameter results analysed for the Jordán river water, which is commonly used for crop irrigation. As can be seen, COD and chloride values are lower than the limits indicated in Colombian regulations (Resolution 1207/2014, Table 1). Similarly to the previous case, the pH parameter remains the same before and after photocatalytic treatment. The nitrate concentration in the Jordán river sample is very low (<1 mg/L), and after the photocatalytic process, the nitrate concentration increased (Figure 8A). However, the final concentration remained very low (<2 mg/L). For chloride ions, the treatment with the coupled photocatalysts does not cause any significant change in its concentration (Figure 8A). A high decrease in chloride was achieved for the dairy industry effluent (>60%), in this case, the chloride concentration could be too low for adsorption on the surface of the coupled systems.

**Figure 9:**
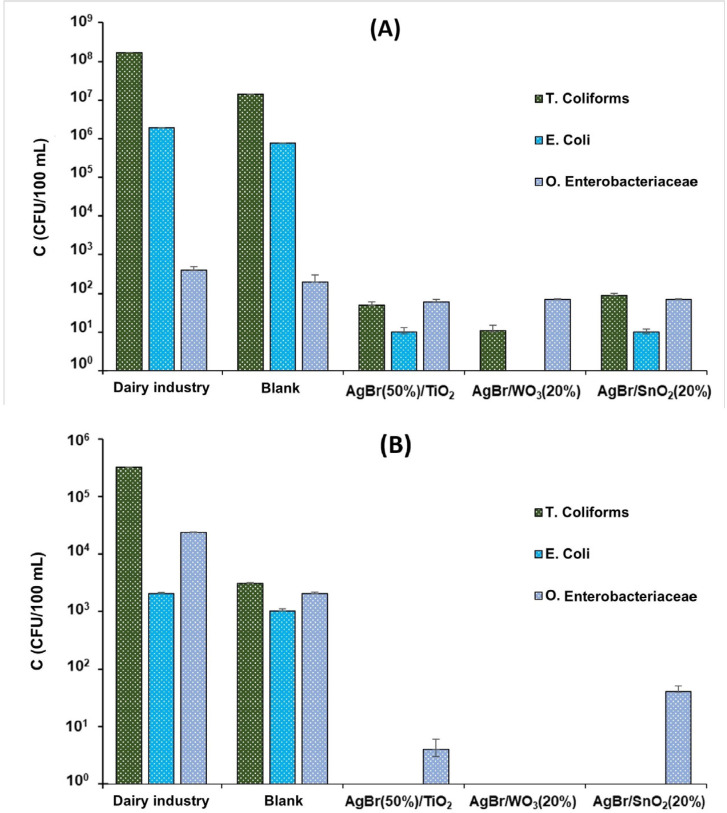
Photocatalytic process on microorganisms from (**A**) dairy industry wastewater; (**B**) Jordán river.

The mechanism of bacterial killing by membrane damage has been extensively studied and reported by different authors [4,5,26,28,83]. The bactericidal effect in the photocatalytic process is mainly associated with the activity of ROS (●OH, H_2_O_2_ and O_2_^•−^), which induce severe damage to bacterial cells. As seen in Table 4, the dairy industry wastewater has a high concentration of *Escherichia coli* and total coliforms. The photolysis process (blank test) on this wastewater achieved a slight decrease in the bacterial load (Figure 9A), mainly due to the bactericidal effect of UV–Vis light, which causes damage to the genetic material of the bacterial cell, thus promoting its death [84]. Meanwhile, the photocatalytic treatment with the AgBr-coupled materials reduced significantly the concentration of both parameters, leading to the complete elimination of *E. coli* with the AgBr/WO_3_(20%) photocatalyst (Figure 9A). However, for other Enterobacteriaceae, none of the composite materials achieved a greater decrease in these microorganisms.

For total and calcium hardness (Figure 8B), only the AgBr/WO_3_(20%) photocatalyst achieved a significant reduction, in contrast to the performance of the AgBr/SnO_2_(20%) sample, where both hardness concentrations remained the same as in the water sample before the photocatalytic process.

Similarly to the dairy industry wastewater, the COD concentration increased after the photocatalytic process (Figure 8C), except for the AgBr(50%)/TiO_2_ sample, where the COD concentration after treatment was almost equal to that observed in the starting water sample. For this photocatalyst, it could be possible that COD increases during the process and then decreases in the final stage, similar to the case of the AgBr/WO_3_(20%) sample with the dairy industrial wastewater. In the case of the real colour, the three photocatalysts achieved a high reduction in this parameter (Figure 8C), with the AgBr/SnO_2_(20%) sample being the most effective in the degradation of coloured pollutants.

Although the concentration of total coliforms and *E. coli* bacteria in the polluted river is high, it is lower compared with the dairy effluent, except for other Enterobacteriaceae. For the Jordán river sample, the AgBr/WO_3_(20%) system showed high disinfectant performance, completely eliminating all bacterial loads. The other two coupled photocatalysts also eliminated total coliforms and *E. coli*, along with a high reduction in other Enterobacteriaceae.

Having completed all these analyses, there are important details to consider: Firstly, as expected, the photolysis process did not reduce the concentration of ions (NO_3_^−^, Cl^−^, Ca^2+^ and Mg^2+^), and no reduction was observed in COD and real colour in this blank test. On the other hand, photocatalytic treatment produced a significant reduction in chlorides and hardness concentrations, at least for the dairy effluent. Moreover, the adsorption of these compounds seems to influence the photoactivity of the coupled materials for pollutant degradation and disinfection. For example, the adsorption of ions by AgBr(50%)/TiO_2_ seems to affect its photoactivity, since there was no decrease in the real colour with the dairy effluent, contrary to that obtained with the Jordán river sample. It is well known that the presence of inorganic ions such as chlorides and nitrates inhibits the formation of hydroxyl radicals and prevents substrates to react with the catalyst surface, thereby reducing the oxidation of organic pollutants [85]. In the case of cations like Ca^2+^ and Mg^2+^, there are reports that indicate a positive, negative or non-existent influence on the photodegradation capacity of the photocatalyst [86,87,88,89]. Nevertheless, this impact depends on different variables, such as pH, cation concentration, substrate and the photocatalyst himself [88]. For dairy industry wastewater, the high adsorption achieved for the coupled materials decreases their photocatalytic activity in the order of AgBr/SnO_2_(20%) > AgBr/WO_3_(20%) > AgBr(50%)/TiO_2_.

On the other hand, in the case of the Jordán river water, the same phenomenon seems to take place with the AgBr/WO_3_(20%) sample, where the adsorption of calcium and magnesium seems to reduce its photoactivity. Instead, the AgBr(50%)/TiO_2_ sample achieved a considerable decrease in COD and real colour in this water sample, possibly due to the low level of ion adsorption. In any case, there is also a high difference in the concentrations of these parameters between the two water samples, which must be considered.

It is interesting to note the inconsistency in the performance observed with AgBr-coupled materials in the treatment of dairy wastewater and river water samples, specifically with those results related to chloride and total hardness contents. As it can be observed in Figure 7 and Figure 8, these quality control parameters significantly decreased after the photocatalytic treatment of the dairy industry effluents; on the contrary, in the case of the Jordán river water, the Cl^−^ and CaCO_3_ values remained almost the same after treatment. This behaviour can be explained taking into account the following:

(i) The water source samples are not comparable; it is because the complexity of the composition of each one is very different. On the one hand, the wastewater came from the outflow on an industrial facility, and on the other hand, the natural water sample was taken from a river highly polluted by different anthropogenic activities, with different industrial and domestic effluents, coming from towns located along the course of the river.
(ii) The starting contents of chlorides and hardness are much higher in the dairy industry sample compared with the river sample.
(iii) The pollutant diversity in the river water could be higher than in that from the dairy industry facility, thus leading to lower effectiveness of the treatment of the first sample.
(iv) In the case of the Cl^−^ content, the final value may be close to the maximum effectiveness of the treatment in the case of the river sample.
(v) The dairy industry sample presented a COD content significantly higher than the river sample, thus indicating high organic load, which can affect behaviour under photocatalytic treatment.

The bacterial concentration in the dairy industry wastewater is higher than in the Jordán river water (except for other Enterobacteriaceae). For the disinfection process, it can be observed that the bactericidal performance of the composite samples seems to be independent of the adsorption or concentration of nitrate, chloride and hardness, because high elimination of bacteria was obtained with the two water sources. However, the results presented in Figure 9 show a potent antibacterial impact on the river water, whereas the bactericidal effect is comparatively modest in the dairy industry wastewater, which can be explained due to the diversity of other Enterobacteriaceae that could be present in these effluents; indeed, in the milk employed as raw ingredient in the dairy industry, it is possible to find about 14 different species of Gram-negative bacteria associated with bovine mastitis illness [90].

The diversity of bacterial species can affect the global effectiveness of photocatalytic treatment. Likewise, as it can be observed in Table 4, the turbidity in the dairy industry wastewater is much higher than in the river water, which can make light penetration inside the reactor difficult, thus affecting photocatalytic activity. Taking into account that photocatalysis is a tertiary treatment, we have recommended, in previous research, the application of flocculation before advanced oxidation technologies to enhance the effectiveness of photocatalytic treatment [29].

Although the bactericidal capacity of the AgBr photocatalyst has been reported, its coupling with other semiconductors enhances its disinfectant performance [91,92]. In this study, the three composite materials exhibited high disinfection capacity. Moreover, in the river sample, complete elimination of *E. coli*, total coliforms and other Enterobacteriaceae was achieved when using AgBr/WO_3_(20%) as the photocatalyst.

According to the results described in the previous section, the AgBr/WO_3_(20%) photocatalyst represents the best alternative for wastewater treatment and is also a potential candidate for the disinfection process.

In heterogeneous photocatalysis it is well known that some properties, such as specific surface area and band gap, are determining factors for the effectiveness of photocatalytic materials. Thus, some authors have found a correlation between surface area and photocatalytic activity [93,94]. Taking into account that the photocatalytic process takes place on the surface, in general, a high specific surface area is a suitable characteristic in the photocatalytic material, leading to a better adsorption of organic compounds and therefore to an improved transformation of these compounds. A high surface area has also been correlated with high antibacterial activity [95]. However, the results obtained in this work showed that a high BET surface area was not a dominant factor for the photocatalytic activity of AgBr/WO_3_(20%); thus, as it can be observed in Table 2, this material presents the lowest S_BET_ value compared with the other solids analysed.

Likewise, a decrease in the energy band gap has been associated with an increase in the photocatalytic activity of solids such as TiO_2_ [96]. Similar results were found in this study, where the photocatalytic materials with the lowest band-gap value (i.e., 2.48 and 2.54 for AgBr(50%)/TiO_2_ and AgBr/WO_3_(20%), respectively) led to the highest bactericidal effect on dairy industry wastewater and Jordán river water.

**Figure 10:**
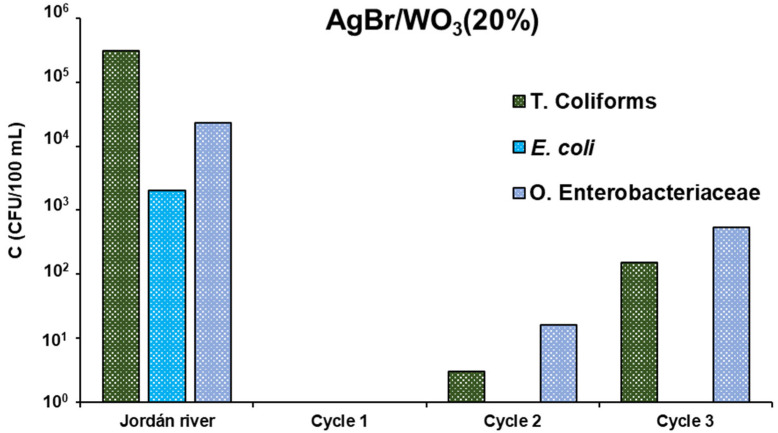
Consecutive cycling runs for disinfection in Jordán river sample with AgBr/WO_3_(20%) photocatalyst.

In order to analyse the reuse of AgBr/WO_3_(20%) material, it was evaluated in successive photocatalytic cycles. These tests were carried out by using this selected photocatalyst in successive cycles with the Jordán river water sample, determining the concentration of total coliforms, *E. coli* and other Enterobacteriaceae after 5 h of treatment. After each test, the photocatalyst was recovered and reused with a new dose of the river sample. Figure 10 shows the results obtained in three consecutive cycles. As can be seen, the composite material completely eliminated *E. coli* bacteria in the three cycles, while for total coliforms and other Enterobacteriaceae, a certain concentration remained in the two last cycles. This indicates that the AgBr/WO_3_(20%) photocatalyst can reach total disinfection only in the first cycle, leaving low concentrations of coliforms and other Enterobacteriaceae in subsequent cycles.

**Figure 11:**
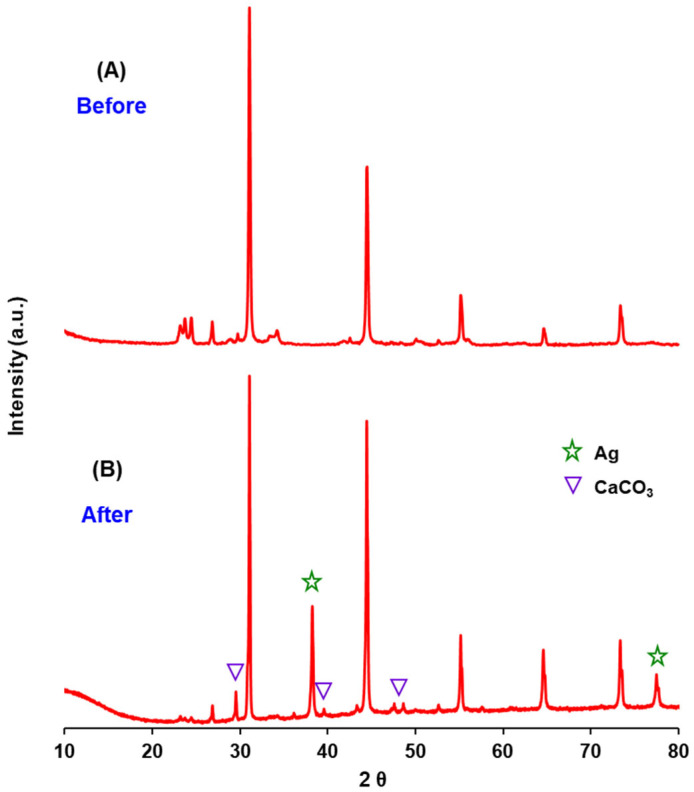
X-ray diffraction patterns of AgBr/WO_3_(20%) before (**A**) and after (**B**) the third cycle of photocatalytic treatment.

In order to analyse the stability of the physicochemical properties of the AgBr/WO_3_(20%) photocatalyst after treatment, XRD and XPS analyses were performed after the third cycle. The diffractograms of the AgBr/WO_3_(20%) sample before (Figure 11A) and after the photocatalytic process (Figure 11B) are quite similar, with some differences. After treatment, the peaks of AgBr and WO_3_ are still clearly observed, with less intense peaks in the case of WO_3_. However, new signals are also observed (indicated by triangles and stars), corresponding to metallic silver (JCPDS No. 01-087-0719) and calcium carbonate (JCPDS No. 01-085-0849). This result indicates that the surface of the composite material undergoes changes due to the adsorption of calcium carbonate and the reduction of Ag^+^.

It has been extensively reported that catalyst deactivation could be due to the blocking of reactive sites on the surface of photocatalysts, which is mainly due to the adsorption of products and intermediates generated by photocatalytic degradation [97,98]. As observed in Figure 10, the AgBr/WO_3_(20%) material suffered successive deactivation. A possible explanation of this decrease in photocatalytic activity in successive reaction cycles could be the accumulation on the photocatalyst surface of species coming from wastewater, such as CaCO_3_. Taking into account these results, it is recommended to explore some methods for photocatalyst regeneration; thus, for example, washing and drying could be employed to clean the material surface.

**Figure 12:**
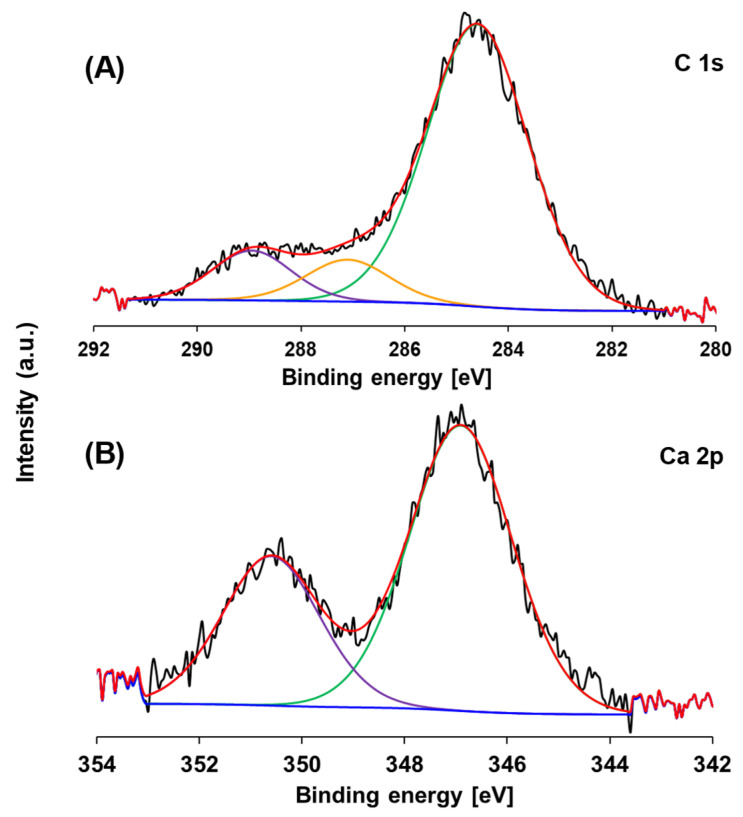
XPS spectra of C 1s and Ca 2p regions of AgBr/WO_3_(20%) after the third cycle of photocatalytic reaction. (**A**) C 1s region and (**B**) Ca 2p region. Blue colour indicates base line, and green, orange and purple colours correspond to the deconvolution of signals performed.

Figure 12 shows the C 1s and Ca 2p XPS regions of the AgBr/WO_3_(20%) photocatalyst after the photocatalytic process. The C 1s spectrum (Figure 12A) can be separated in three components, with peaks located at 284.6 ± 0.1, 287.1 ± 0.1 and 289.0 ± 0.1 eV being assigned to adventitious carbon, and the C–O–C and O–C=O bonds of CaCO_3_, respectively. The high-resolution spectrum of Ca 2p (Figure 12B) presents two peaks at 346.9 ± 0.1 and 350.6 ± 0.1 eV, assigned to Ca 2p_3/2_ and Ca 2p_1/2_, respectively. The presence of calcium and the new signals in the C 1s spectrum, corresponding to C–O–C and O–C=O bonds, together with the XRD analysis, confirm the existence of calcium carbonate on the surface of AgBr/WO_3_(20%) after the photocatalytic process.

**Figure 13:**
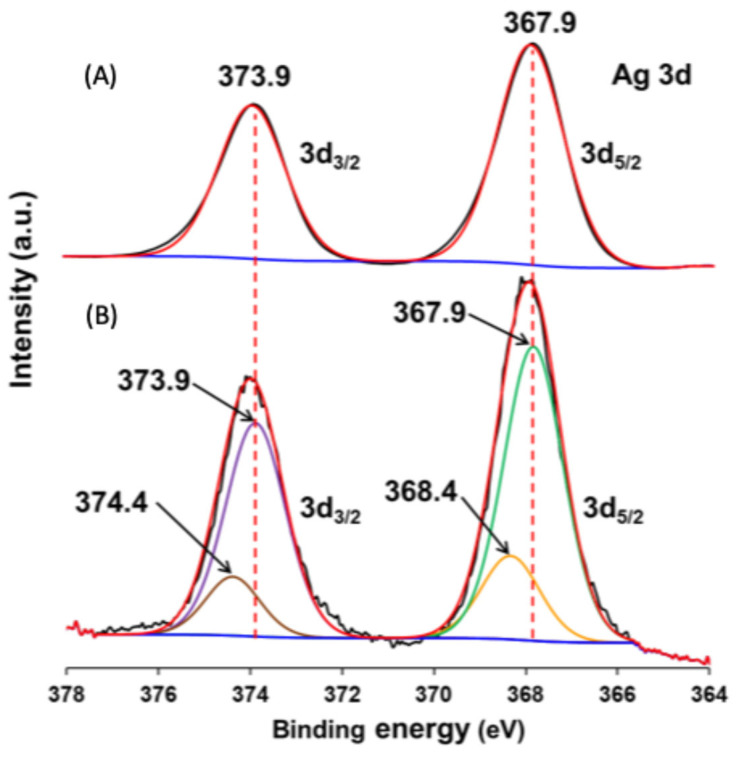
XPS spectra for Ag 3d region of AgBr/WO_3_(20%) before (**A**) and after (**B**) the third cycle of photocatalytic reaction. Blue colour indicates base line, and green, orange and brown colours correspond to the deconvolution of signals performed.

On the other hand, for the Ag 3d region (Figure 13), differently from the spectrum obtained with the initial AgBr/WO_3_(20%) sample (Figure 13A), the peaks for Ag 3d_5/2_ and Ag 3d_3/2_ can be divided into two components. The Ag 3d_5/2_ signal is divided into two peaks, at 367.9 ± 0.1 and 368.4 ± 0.1 eV, and the Ag 3d_3/2_ signal is also divided into two peaks, at 373.9 ± 0.1 and 374.4 ± 0.1 eV. Consistently with previous XPS analysis, the peaks at 367.9 ± 0.1 and 373.9 ± 0.1 eV are assigned to the Ag^+^ of the AgBr present in AgBr/WO_3_(20%). The remaining two signals, with higher binding energies (368.4 ± 0.1 and 374.4 ± 0.1 eV), are assigned to Ag^0^ [99,100].

In general, by XPS and XRD analyses, the presence of metallic silver on the surface of the coupled photocatalyst is confirmed. One could question the following: If Ag^+^ is reduced and there is no silver in the medium, what happens to Br^−^? Is it oxidised to Br_2_? Although this hypothesis is plausible, it does not seem to be the case, since the stability of the AgBr(50%)/TiO_2_ coupled sample against photocorrosion after 120 min of illumination has been studied; the results obtained for these samples are presented in the reference [38], and it is evident that the AgBr(50%)/TiO_2_ sample, after a prolonged period of illumination, practically does not release silver into the medium, although it does release bromide ions.

The results mentioned above indicate that during the photocatalysis process, the AgBr/WO_3_(20%) material undergoes changes in its properties, becoming a system with a different composition. There is likely to be some leaching for AgBr, where Ag^+^ is reduced and deposited on the photocatalyst surface, while the adsorption of calcium ions takes place through the deposition of calcium carbonate on the coupled system.

Regarding the generation of metallic silver on the surface of AgBr/WO_3_(20%) material, it is worth to mention a previous work conducted by our research group where the photodegradation of RhB in aqueous solution with this coupled material was studied. In the work, the photocatalyst was also analysed by XPS after dye treatment, which slowed down some changes in its composition, although with different results. In the work [36], XPS analysis revealed a shift in the Ag 3d peaks towards lower binding energies, indicating the possible existence of Ag_x_O and/or Ag_2_CO_3_.

All these pieces of information suggest that AgBr/WO_3_ in the coupled system is not a photostable material, undergoes compositional changes during the photocatalytic process, and depending on the type of substrate treated, could generate species such as Ag, Ag_x_O and Ag_2_CO_3_.

As prospective work, further studies about the immobilisation of the AgBr-coupled materials are recommended; it is important to take into account that under real conditions, in the application of heterogeneous photocatalysis on an industrial or large scale, the photocatalytic material must always be recovered and separated from the water after treatment.

## 4. Conclusions

Different photocatalytic materials based on AgBr coupled with TiO_2_, WO_3_ or SnO_2_ were successfully synthesised. The material prepared by coupling AgBr with WO_3_ showed the best photocatalytic performance in the treatment of wastewater from a dairy industry facility and from a polluted river. After treatment with this material, it was possible to decrease the values of water quality control parameters such as chlorides, colour and hardness, along with the total elimination of *E. coli*, total coliforms and other Enterobacteriaceae.

This research study represents an interesting starting point for conducting further studies about the potential of photocatalytic treatment using AgBr-based coupled systems for dairy industrial effluents, focused on the reduction in the negative environmental impact of these effluents. Additionally, these coupled materials could represent an opportunity for the treatment of river water, before using the latter in crop irrigation. However, it is very important to take into account that photocatalysis is a tertiary treatment, which should be combined with different primary methods in order to improve its global effectiveness in water treatment.

This study also highlights the need to simultaneously study several water quality parameters at the same time, such as hardness, ions, coloured pollutants, pH, microorganisms, etc., since the development of the photocatalytic process for the abatement of a particular pollutant or microorganism could be different depending on the concentration of the mentioned parameters.

In order to achieve the effective removal of COD and nitrates, it will be necessary to apply additional processes in combination with photocatalysis, and considering the highest photocatalytic performance observed with the use of AgBr/WO_3_(20%) material, it is very important to conduct further research on some alternatives to improve its stability during photocatalyst recycling.

The identification of the intermediate products generated during the photocatalytic treatment and its management, the photocatalyst amount and the light intensity are also important concerns to consider in further research.

## Figures and Tables

**Table 1:** Regulations for water quality parameters.

Water Quality Parameter	Resolution 631/2015	Resolution 1207/2014
COD (mg/L O_2_)	450.0	450.0
pH	6.0–9.0	6.0–9.0
Nitrates (mg/L)	Analysis *	5.0
Chlorides (mg Cl^−^/L)	500.0	300.0
Total hardness (mg CaCO_3_/L)	Analysis *	NR **
Calcic hardness (mg CaCO_3_/L)	Analysis *	NR **
Real colour (absorbance measured at wavelength of 436 nm)	Analysis *	NR **
*E. coli* (CFU/100 mL)	NR **	NR **
Total coliforms (CFU/100 mL)	NR **	NR **
Other Enterobacteriaceae (CFU/100 mL)	NR **	NR **

* Analysis: No limits are established in the regulations, and only the analysis and the reported value are requested. ** NR: Not required by the regulations.

**Table 2:** Selected physicochemical properties for AgBr-coupled photocatalysts.

Photocatalyst	Crystallite Size D (nm) AgBr(200)	S_BET_ (m^2^/g)	Band Gap (eV)
AgBr	175.5	<1	2.60
TiO_2_	-	91.0	3.10
SnO_2_	-	111	3.92
WO_3_	-	30.2	2.80
AgBr(50%)/TiO_2_	242.4	23.4	2.48 (*)
AgBr/SnO_2_(20%)	149.9	12.0	2.60 (*)
AgBr/WO_3_(20%)	107.1	6.60	2.54 (*)

(*) Pseudo band-gap values obtained for the coupled materials.

**Table 3:** Band positions estimated for AgBr, TiO_2_, SnO_2_ and WO_3_.

Semiconductor	Electronegativity	Eg	VB Position	CB Position
	**(X)**	**(eV)**	**(eV) vs. NHE**	**(eV) vs. NHE**
AgBr	4.42	2.60	1.22	−1.38
TiO_2_	5.81	3.10	2.86	−0.24
WO_3_	6.33	2.80	3.23	0.43
SnO_2_	6.25	3.92	3.71	−0.21

**Table 4:** Physicochemical and microbiological analyses of dairy industry wastewater and Jordán river samples.

Quality Control Parameter	Dairy Industry	Jordán River
COD (mg/L)	117,500	23
pH	6.75	6.68
Nitrates (mg/L)	97	0.5
Chlorides (mg/L)	597	27
Total hardness (mgCaCO_3_/L)	1320	235
Calcium hardness (mgCaCO_3_/L)	825	144
Real colour (absorbance measured at wavelength of 436 nm)	10,290	207
*E. coli* (CFU/100 mL)	2 × 10^6^	2.0 × 10^3^
Total coliforms (CFU/100 mL)	1.8 × 10^8^	3.1 × 10^5^
Other Enterobacteriaceae (CFU/100 mL)	4.0 × 10^2^	2.3 × 10^4^

## Data Availability

Data will be made available upon request.
